# Mapping the Use of Information and Communication Technologies in Health Interventions for Older Adults: A Rapid Review

**DOI:** 10.1007/s10823-026-09572-1

**Published:** 2026-05-13

**Authors:** Angelica Baptista Silva, Maria Tereza Leal, Sergio Ricardo Ferreira Síndico, Mercedes Serrano-Miguel, Helena Maria Almeida Macedo Loureiro, Loureiro

**Affiliations:** 1 Escola Nacional de Saúde Pública Sérgio Arouca, Fundação Oswaldo Cruz, Rio de Janeiro, Brazil; 2 Unitat de Formació i Recerca de Treball Social. Facultat d’Educació. Barcelona, Universitat de Barcelona, Barcelona, España; 3 Instituto de Comunicação e Informação Científica e Tecnológica em Saúde. Biblioteca da Saúde da Mulher e da Criança, Fundação Oswaldo Cruz, Rio de Janeiro, Brazil; 4 Escola Superior de Saúde, Universidade de Aveiro, Aveiro, Portugal

**Keywords:** Aging, Health Services, Health Professionals, Information and Communication Technologies, eHealth

## Abstract

This rapid review mapped how information and communication technologies (ICTs), particularly digital health applications, are being used within healthcare services for adults aged 55 years and older. Following the Joanna Briggs Institute methodology and guided by the Population–Concept–Context framework, studies published between 2016 and 2024 were identified and reported in accordance with the PRISMA extension for scoping reviews, based on a previously published protocol registered on the Open Science Framework. Nineteen studies were included after critical appraisal. The findings indicate that ICT use is most frequently associated with improved accessibility and coordination of healthcare services, support for health professionals’ training and care management practices, and positive health-related outcomes for older adults, particularly in the domains of mental health, social participation, and self-care. However, the evidence remains heterogeneous and often limited in its reporting of design and implementation processes, highlighting the need for further context-sensitive research that addresses ethical, equity, and implementation considerations in the use of digital health technologies for older populations.

**Supplementary Information:**

The online version contains supplementary material available at 10.1007/s10823-026-09572-1.

## Background

The expansion of information and communication technologies (ICTs) in health has increasingly reshaped the organization of health services and the strategies used to promote health across populations. Digital health applications, in particular, have been widely disseminated as tools to support health monitoring, self-care, and continuity of care. However, despite their rapid proliferation, there is a recognized lack of consolidated evidence regarding their use and scope, as only a limited proportion of health applications have been systematically investigated, and the quality of available evidence remains uneven (Diao et al., 2022).

Within this broader digital health context, population ageing represents a particularly relevant field of application. As a result of demographic transitions, increased healthy life expectancy has led to the emergence of a growing group of adults over 60 years of age who remain active and engaged in social and working life (Mason et al., 2022). In gerontology and public health, later adulthood is increasingly understood as a heterogeneous life stage that follows middle adulthood but does not necessarily imply dependency or functional decline, challenging more traditional age-based representations (Cao et al., 2022).

One relevant challenge in this context concerns how digital health technologies are designed, implemented, and used by adults in later stages of the life course. Age-specific forms of digital and health literacy, together with differences in prior technological experience, accessibility, and usability, may limit the effective uptake of digital health applications among older adults. When such specificities are not adequately considered, digital tools risk reinforcing existing inequalities rather than supporting meaningful engagement in health promotion and care processes *(*Shi et al., 2024). Interventions that accompany transitional phases in later adulthood—such as the period approaching retirement—have therefore been identified as particularly relevant for supporting health promotion and active ageing (Loureiro et al., 2015).

In institutional and policy contexts, chronological age thresholds—such as the benchmark of 65 years commonly used by the World Health Organization—are frequently applied for administrative purposes, including retirement eligibility and access to social benefits. Nevertheless, these thresholds are best understood as contextual conventions rather than intrinsic indicators of health status, functionality, or dependency. Contemporary gerontological perspectives emphasize ageing as a socially constructed and heterogeneous process, shaped by social, cultural, and occupational trajectories rather than by age alone (Schneider & Irigaray, 2008).

Against this background, the objective of this rapid review was to map the use of information and communication technologies—particularly digital health applications—within healthcare services for adults aged 55 years and older. This age group often represents a transitional phase between midlife and later adulthood and is commonly associated with changes related to work, retirement preparation, and everyday life. The review aimed to identify how these technologies are being applied in healthcare contexts, the types of practices and interventions described, and the areas of health promotion addressed. Given the broad and exploratory nature of the topic, a rapid review approach was considered appropriate to provide a timely mapping of existing evidence without aiming to assess the effectiveness of specific interventions.

This review was conducted in accordance with a previously published protocol (Silva et al., 2024), developed following the Joanna Briggs Institute methodology for scoping reviews and guided by the Population–Concept–Context framework. The protocol was prospectively registered on the Open Science Framework (OSF 10.17605/OSF.IO/PQB3R) ensuring transparency and alignment between planned and reported methods. Accordingly, this review addressed the following research question: How are digital health applications being used within healthcare services for adults aged 55 years and older?

## Methods

**Fig. 1:**
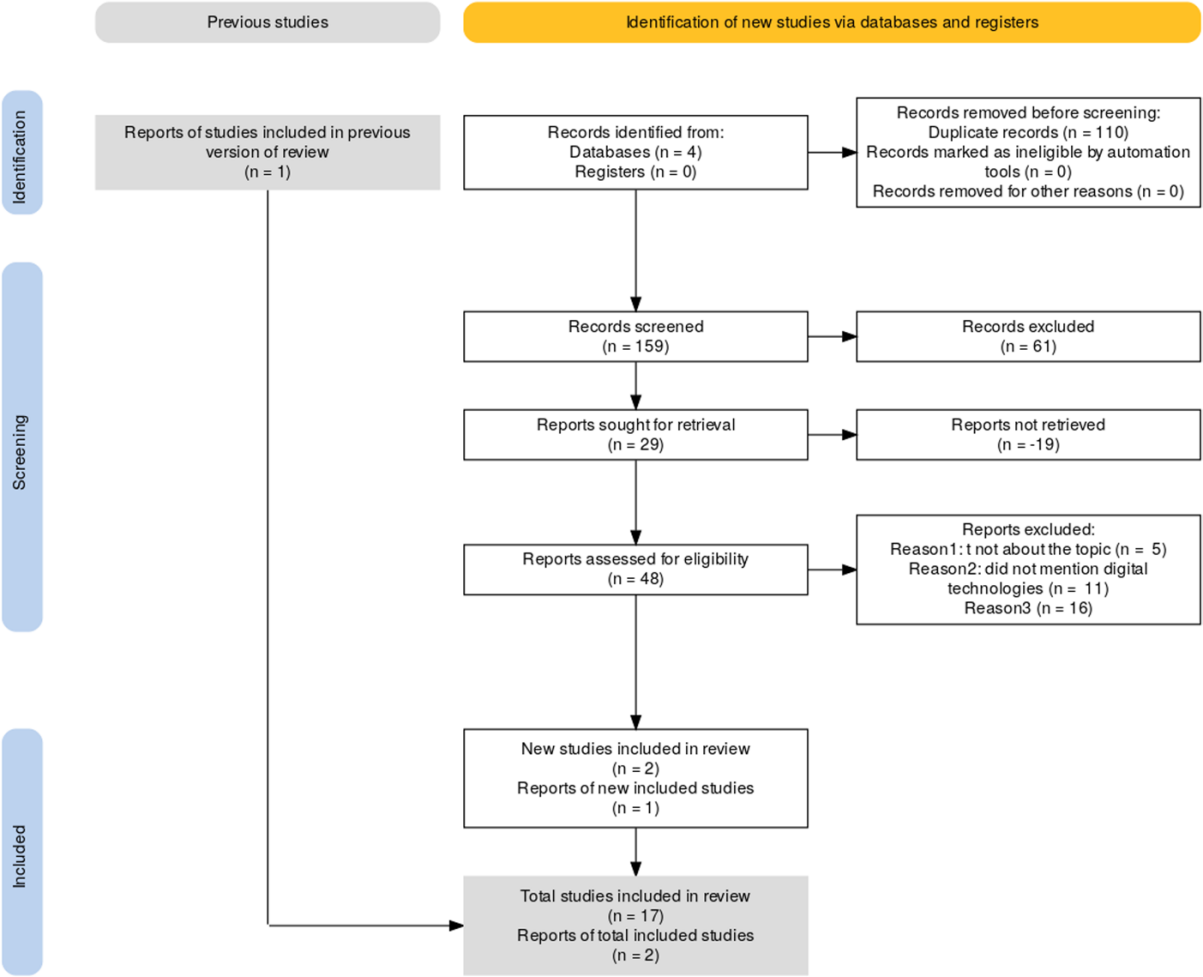
The PRISMA diagram shows the numbers of reports retrieved and the flow through the stages of the scoping review

This review was conducted using the Joanna Briggs Institute (JBI) methodological framework for scoping reviews, guided by the Population–Concept–Context (PCC) approach. As specified in the published protocol (Silva et al., 2024), a scoping review methodology was selected because of the broad and exploratory nature of the research question and the absence of an intention to assess the effectiveness of specific interventions. In the present study, this methodological approach was finally implemented as a rapid review in order to provide a timely and focused mapping of the available evidence. The review protocol was developed in accordance with the Preferred Reporting Items for Systematic Reviews and Meta-Analyses Protocols (PRISMA-P), and the reporting of results followed the PRISMA extension for Scoping Reviews (PRISMA-ScR) checklist, with 159 studies identified in the initial screening, ultimately yielding 17 studies eligible for analysis and discussion (Fig. 1).

### **Source**

The authors adapted from Haddaway, N. R., Page, M. J., Pritchard, C. C., & McGuinness, L. A. (2022). PRISMA2020: An R package and Shiny app for producing PRISMA 2020-compliant flow diagrams, with interactivity for optimized digital transparency and Open Synthesis Campbell Systematic Reviews, 18, e1230. 10.1002/cl2.1230.

### Participants

This review considered studies involving healthcare services, health professionals, and older adults aged 55 years and older.

## Concept

This review examined the concept of Information and Communication Technologies (ICTs) in health, focusing on studies that described the use of applications for mobile phones and other digital technologies adopted within public healthcare services. Studies were excluded if they focused exclusively on clinical disease treatment and did not address health promotion.

## Context

This review considered studies published between 2016 and 2024 that examined the use of mobile health applications to support practices conducive to healthy living among older adults within primary healthcare services. This period was selected to capture evidence produced before and after the COVID-19 pandemic, using 31 December 2019—the date on which the World Health Organization received the first official notification of the outbreak—as a key temporal milestone.

### Inclusion Criteria

This review included experimental and quasi-experimental study designs, analytical observational studies, and descriptive observational studies. Qualitative and mixed-method studies were also eligible for inclusion. In addition, secondary studies (e.g., systematic reviews), discussion papers, and policy documents relevant to the review question and meeting the inclusion criteria were considered. Letters, editorials, and protocols were excluded.

The search strategy aimed to identify both published and unpublished primary studies, reviews, policy documents, and opinion papers. An initial limited search was conducted in EMBASE (Elsevier) and MEDLINE (PubMed) to identify studies relevant to the topic. A comprehensive search strategy was developed with the support of an expert librarian, using key terms and index terms identified from the titles and abstracts of relevant studies. This strategy was subsequently adapted for each database consulted. In addition, the reference lists of all included studies were screened to identify further relevant publications.

Studies published in English, Portuguese, and Spanish were included. Sources of unpublished and grey literature included Web of Science, Scopus, Thesaurus, and PROSPERO, as well as governmental and non-governmental documents such as country reports, policies, and regulations related to the topic.

The search terms were organized into four thematic groups aligned with the scope and objectives of the review: older adult health, digital technologies, mental health, and retirement. Each group was operationalized in the search strategy through a set of related terms, including elderly care, health of the elderly, services for the aged, individuals aged 80 and over, older adults, Internet and Internet use, dashboard systems, platforms and online platforms, digital media, digital health, artificial intelligence, mental health and related services, teletherapy, follow-up, and retirement-related terms such as retired and retire.

A specialized health librarian was responsible for identifying and selecting the most appropriate descriptors to reflect the review objectives and for implementing them in the selected databases. The search strategy applied Boolean operators, using OR to combine synonymous or related terms within each category and AND to link different categories. Additionally, the librarian adapted and translated the search field tags—such as title, abstract, and keywords—to ensure compatibility with the specific syntax requirements of each database consulted.

This review may be affected by publication bias, since most of the studies included reported positive results regarding feasibility, usability, or effectiveness. Evidence in the literature indicates that studies with favorable outcomes are more likely to be published, which can result in an inflated perception of the effectiveness of these types of interventions when assessed according to the reported outcome measures (Berry et al., 2025; Boutron et al., 2019).

This review included a range of study designs, encompassing experimental and quasi-experimental studies, as well as analytical and descriptive observational research. Qualitative and mixed-method studies were also considered. Furthermore, relevant secondary sources, such as systematic reviews, discussion papers, and policy documents that aligned with the review questions and met the inclusion criteria, were assessed for inclusion. Studies in the form of letters, editorials, and protocols were excluded.

We considered it relevant to include secondary studies in this review because these studies help to locate relevant literature in a field, identify gaps, and guide new research efforts (Felizardo et al., 2020). Systematic reviews have emerged in response to the exponential growth of available information, aiming to provide healthcare professionals with a rigorous analysis that distinguishes findings relevant for clinical practice. They represent a cornerstone of evidence-based medicine, with two critical steps—comprehensive literature searching and careful appraisal of studies—forming the basis for informed medical decisions, particularly regarding diagnosis, treatment, and prognosis (Villasís-Keever et al., 2020).

### Study Selection and Screening

Following the search, all identified citations were uploaded to Rayyan (https://rayyan.ai/reviews/921238/) and duplicates were removed. After a pilot screening, titles and abstracts were independently screened by two reviewers to assess eligibility according to the inclusion criteria. Potentially relevant studies were retrieved in full, and their citation details were imported into Rayyan.

The full texts of selected studies were independently assessed against the inclusion criteria by two reviewers. Reasons for exclusion at the full-text stage were recorded and reported in the review. Any disagreements between reviewers at any stage of the selection process were resolved through discussion or, when necessary, by consultation with a third reviewer. The results of the search and selection process are reported in the final review and presented in a PRISMA flow diagram.

Data were extracted from the included studies by two independent reviewers using a data extraction tool adapted from the Joanna Briggs Institute (JBI) data extraction framework. Extracted data included details on participants, concept, context, study design, and key findings relevant to the review question. A draft version of the data extraction tool was developed and is provided as supplementary material. The extraction tool was iteratively refined during the data extraction process as necessary. Any disagreements between reviewers were resolved through discussion or, when required, by consultation with a third reviewer.

To support the systematization of results and language refinement, the authors used ChatGPT (version 4o; https://chatgpt.com/) and Grammarly (https://app.grammarly.com/). These tools were used exclusively for organizational and language-related purposes and did not influence data interpretation or study selection.

The methodological quality of the included studies was assessed independently by two reviewers using the appropriate JBI Critical Appraisal Tools, according to each study’s design (https://jbi.global/critical-appraisal-tools).

## Results

The methodological quality of the included studies was assessed through the application of the appropriate JBI critical appraisal tools, using an evaluation grid tailored to the study designs included in the review. This appraisal covered 4 quasi-experimental studies, 5 randomized controlled trials, and 10 observational studies. Inter-reviewer agreement was high (Cohen’s kappa = 0.85), and any disagreements in appraisal were resolved by consensus (Pearson et al., 2024).

A total of 19 studies were included in the review. Of these, 31% were conducted in China, with the remaining studies distributed across several other countries. The small number of studies resulting from the synthesis, in addition to the systematization method is related to the specificity of the topic and the still limited academic production regarding the intersection of ICTs and healthcare for older adults. In a scoping review on ICTs for self-care among older adults with chronic diseases (≥ 55 years), only 31 studies were included out of 1,149 initial records (2.7%), indicating limited specific research and many studies not directly focused on older adults (Zaman et al., 2022). A review on e‑health/m‑health for health promotion and primary prevention in people aged 50 + identified 45 studies over 10 years, highlighting that use “outside formal programs” is scarcely investigated (Kampmeijer et al., 2016). A scoping review of digital technologies for health promotion and prevention in older adults identified 98 studies (90 primary, 8 reviews) with low overlap among the primary studies, both across the 8 relevant systematic reviews and between the reviews and our own search. This reflects a heterogeneity of approaches and limited thematic specificity, suggesting that the field is still poorly consolidated (De Santis et al., 2023).

One study conducted a cross-national secondary analysis using data from two national longitudinal surveys—the 2018 and 2020 *Health and Retirement Study* (HRS) in the United States and the 2018–2019 and 2020 waves of *Understanding Society* (UKHLS) in the United Kingdom—examining inter-household contact and mental well-being among adults (Hu & Qian, 2021). In contrast, one study implemented a multi-country digital intervention, evaluating the impact of a digital coaching programme on physical activity, mental well-being, and social participation among adults approaching retirement in Italy and the Netherlands (Santini et al., 2023).

Extending this cross-national perspective, three additional studies examined evidence from more than two countries. One study addressed digital exclusion across China, the European Union, and Mexico through a comparative analysis of multiple longitudinal surveys, including the *Health and Retirement Study* (HRS), the *English Longitudinal Study of Ageing* (ELSA), the *Survey of Health*,* Ageing and Retirement in Europe* (SHARE), the *China Health and Retirement Longitudinal Study* (CHARLS), and the *Mexican Health and Aging Study* (MHAS) (Lu et al., 2022). Another study analysed data from countries within the European Union and Israel, investigating the reciprocal association between internet use and cognitive functioning over a two-year period using a large multinational sample of older adults (Kamin & Lang, 2020). In addition, a technical report from the WeLar Project assessed the impact of digitalisation on older workers, with a particular focus on the propensity for early retirement across 27 European Union countries (Nguyen-Thi et al., 2024).

Table 1 summarizes the main characteristics of the studies included in this review, highlighting the diversity of technologies, study designs, populations, and intervention contexts examined across different territories and cultural settings. The table presents key information on authorship and institutional affiliation, year of publication, study design, study population, and the context in which digital health technologies were applied. As shown, the reviewed studies encompass a wide range of digital interventions—including mobile applications, internet-based platforms, digital coaching systems, and technology-supported training programmes—implemented within healthcare services, workplace settings, and community contexts. This heterogeneity reflects the varied ways in which information and communication technologies are being used to promote health and well-being among older adults across different social and institutional environments.

**Table 1:** Characteristics of the included studies

Author citation and affiliation	Date of publication	Study design	Study population	Context of intervention
Loveys K, Sagar M, Pickering I, Broadbent E. Department of Psychological Medicine, University of Auckland, New Zealand	November, 2021	Randomized Controlled Trial	*n* = 30. Female = 24 Age > 18y	Digital Humans to deliver a remote loneliness and stress intervention to adults who were at greater risk of developing severe illness if they contracted COVID-19, and as a result, they were asked by the local New Zealand Government to self-isolate to a greater degree during the pandemic.
Hu Y and Qian Y. Department of Sociology, Lancaster University, Lancaster, United Kingdom, Department of Sociology, University of British Columbia, Vancouver, BC, Canada.	July, 2021	Observational	*n* = 6539, 1391(US), 5148(UK). Female = 3460 Age > 60y	Measure inter-household contact and mental well-being before and after pandemics, comparing virtual and face-to-face interactions.
Lu, XR and Yao, Y and Jin, YZ. Department Global Health Peking University, Beijing, China.	December, 2022	Observational	*n* = 13,457 Female = 6867 Age > 45y	Investigate the association between digital exclusion and functional dependency among older adults from high-income countries (HICs) and low- and middle-income countries (LMICs), analyzing national longitudinal studies performed in China, European Union and Mexico.
Braun L, Titzler I, Terhorst Y, Freund J, Thielecke J, Ebert DD et al. Department of Clinical Psychology and Psychotherapy and Department of Research Methods, University of Ulm, Department of Clinical Psychology and Psychotherapy, Friedrich-Alexander-University of Erlangen-Nürnberg, GET.ON-Institute, Germany. Department of Clinical, Neuro- & Developmental Psychology, VU University, Netherlands.	September, 2020	Randomized Controlled Trial	*n* = 340 Female = 196 Age 40 > = 60y	Normal treatment was compared to an e-coach with 6 programs related to diabetes, alcohol consumption, insomnia, depression, panic syndrome, and stress. 6–8 weekly standard modules with a duration of 30–60 min per module with multimodal content. After completion of the training phase, participants were followed up for 12 months by their individual eCoach with a monthly exchange via telephone or internal messaging function to promote the knowledge transfer into everyday life.
Yinzi J, Mingxia J, Xiaochen M. Department of Global Health, School of Public Health, Peking University, Department of Public Health, Shihezi University School of Medicine, Center for Health Development Studies, Peking University, China.	July, 2022	Observational	*n* = 13,457 Female = 6867 Age > = 45	Use a Chinese longitudinal survey to investigate whether desktop and cellphone ownership might be independently associated with decreased cognitive decline in mid-life and older adulthood; and to examine the combined effect of both of the digital devices on cognitive decline.
Nguyen-Thi TU, Barslund M, Ivkovic I, Milinkovic A & Tobback I. Belgrade University, Serbian and partners.	January 2024	Observational	No info about gender and the total sample. Age > 50y	They assess the impact of digitalization by analyzing the occupation-specific employment retention rates of older workers the EU-27 countries (AT, BE, BG, CY, CZ, DE, DK, EE, EL, ES, FI, FR, HR, HU, IE, IT, LT, LU, LV, MT, NL, PL, PT, RO, SE, SI and SK) and examine the 5-year occupational retention rates of 50–54 and 55-59-year olds and relate them to their exposure to ICT capital (at ages 50–54 and 55–59). Research data covers from 2006 to 2019.
Byrne, KA and Anaraky, RG and Dye, C and Ross, LA and Madathil, KC and Knijnenburg, B and Levkoff, S. Department of Psychology, Department of Human-Centered Computing, Department of Civil Engineering, Department of Industrial Engineering, Clemson University, College of Social Work, University of South Carolina United States.	August, 2021	Observational	*n* = 4315 Female = 2616 Age > 50y Race was categorized as non-Hispanic Black/African American, other racial/ethnic group (including Asian, American Indian/Alaskan Native, and Native Hawaiian/Pacific Islander, and individuals who identified as “other”), or non-Hispanic White/Caucasian. The categorical variable rurality was operationalized as rural, suburban, or urban.	They investigated racial and rural-urban differences in the relationship between social technology use and loneliness from 2016 survey of the Health and Retirement Study (HRS), a nationally representative longitudinal study of Americans that includes demographics, health, and cognitive measures.
Ma Y, Liang C, Gu D, Zhao S, Yang X, Wang X. Anhui University of Traditional Chinese Medicine, China.	February, 2021	Observational	*n* = 1020 Female = 499 Age > 55y	They analyzed the results of the formal questionnaire respondents of the Bengbu social security office to measure the effect of older workers’ social media usage at work on their work ability (related to both physical and mental health) and thus their willingness to delay retirement.
Neil-Sztramko SE, Coletta G, Dobbins M, Marr S. School of Nursing, School of Kinesiology and Division of Geriatrics, McMaster University, Canada.	April, 2020	Quasi-experimental	*n* = 32 Female = 20 Age > 60y	Five modules, with 1 education session left free for review and participant-specific questions. The first week of classes focused on learning the basic features of an iPad and locating the variety of available apps. In subsequent weeks, participants learned how to use specific applications, including using the internet, taking and viewing photographs, sending and receiving emails, and using basic apps). The modules were accompanied by a participant workbook that included session content, homework, and additional information to help the participants learn the material.
Kamin ST, Lang FR. Institute of Psychogerontology, Friedrich-Alexander-University Erlangen-Nuremberg (FAU), Germany.	August, 2018	Observational	*n* = 29,576 Female = 17,450 Age > 50 They excluded participants who reported diagnoses of cognitive impairments or other neurological problems (Alzheimer’s disease, dementia, stroke, and Parkinson disease) at one of the measurements.	The study included longitudinal data from 2013 (Wave 5; T1) and 2015 (Wave 6; T2). It´s representative data across 14 countries (Austria, Germany, Sweden, Spain, Italy, France, Denmark, Switzerland, Belgium, Israel, Czech Republic, Luxembourg, Slovenia, and Estonia) from the Survey of Health, Ageing and Retirement in Europe (SHARE).
Kaminsky AM, Gorroochurn P, Hark LA, Cioffi G, Liebmann JM, Sharma T, et al. Department of Ophthalmology, Columbia University, Vagelos College of Physicians and Surgeons; Edward S. Harkness Eye Institute, Columbia University Irving Medical Center; Department of Biostatistics, Columbia University Mailman School of Public Health; Kresge Eye Institute (Y.S.K.), USA Inquiries to Lisa Hark, Columbia University Irving Medical Center, Edward S. Harkness Eye Institute, New York, New York; Center for Health Outcomes, Policy, and Economics (L.T.P.), Rutgers University Ernest Mario School of Pharmacy, Piscataway, New Jersey, USA; Department of Health Services, Policy & Practice (E.J.), Brown University School of Public Health, Providence, Rhode Island; Westat Public Health and Epidemiology Practice (S.S.), Rockville, Maryland; Massachusetts Eye and Ear Infirmary (D.S.F.), Harvard Medical School, Glaucoma Service, Boston, Massachusetts, Wayne State University School of Medicine, Detroit, Michigan, USA.	January, 2023	Randomized Controlled Trial	*n* = 703 Female = 245 Age > 40y	A group approach whose focus was improving quality of life and digital inclusion conducted in either the community room at the NYCHA development or the Department for the Aging (DFTA) Senior Centers. Recruitment in Harlem and Washington Heights neighborhoods targeted high-risk individuals living at or below the NYC.gov poverty measure. Participants made eye health screenings, visual acuity with correction, intraocular pressure measurements, and fundus photography. Participants with VA 20/40 or worse, IOP 23–29 mm Hg, or an unreadable fundus image failed the screening and were scheduled for an optometric examination at the same location; those with an abnormal image were referred to ophthalmology.
Liu Q, Pan H, Wu Y. School of Public Administration, Hunan Normal University, Department of Sociology, Zhejiang University, Hangzhou 310,058, China	August, 2020	Observational	*n* = 40,789 Female = 20,856 Age > 45y	They observed three waves of panel data (2011, 2013, and 2015) from the CHARLS. Respondents in 10,257 households from 450 villages/urban communities in 150 counties/districts across 28 provinces in China to test 4 hiphotesis: (1) Migration would increase depression. (2) Migration would increase the frequency of internet use. 3)Internet use would mediate the relationship between migration and depression and increase the degree of social participation. 4)Social participation would mediate the relationship between internet use and depression among middle-aged and older people in China.
Du X, Liao J, Ye Q, Wu H. Taikang Tongji (Wuhan) Hospital, Tongji Hospital, Tongji Medical College, Huazhong University of Science and Technology, School of Medicine and Health Management, Tongji Medical College, Huazhong University of Science and Technology, Wuhan, China.	August 2023	Observational	*n* = 17,676 Female = 9243 Age ≥ 45y	They examined the CHARLS dataset to investigate the association between internet use and depression. Respondents were asked about the frequency of their internet use and about 8 categories: chatting, watching news, watching videos, playing games, financial management, mobile payment, WeChat, and moments.
Lara J, O’Brien N, Godfrey A, Heaven B, Evans EH, Lloyd S, et al. Human Nutrition Research Centre, Newcastle University; Institute of Cellular Medicine, University Institute for Ageing, Institute of Health and Society, Institute of Neuroscience, Centre for Ageing & Vitality (CAV), Newcastle University; Department of Applied Sciences, Faculty of Health & Life Sciences, Northumbria University; UKCRC Centre for Translational Research in Public Health; Centre for Oral Health Research, Newcastle upon Tyne, People Services, Redcar & Cleveland Borough Council, Redcar; Centre for PublicPolicy and Health, School of Medicine, Pharmacy and Health, Durham University Queen’s Campus, Stockton on Tees; Health and Social Care Institute, School of Health and Social Care, Teesside University, Middlesbrough, Tees Valley; Centre for Diet and Activity Research (CEDAR), MRC Epidemiology Unit, University of Cambridge, Cambridge, United Kingdom. Department of Psychiatry & Behavioral Sciences, University of Texas HSC, Houston, Texas, United States of America.	July, 2016	Randomized Controlled Trial	*n* = 75 Female = 57 Age > 59y	Health Improvement Specialists—involved in workplace health activities via the Northeast Better Health at Work award —facilitated contact with employers who in turn supported participant recruitment. The intervention was based on the Health Action Process Approach, a theoretical framework recognising the importance of planning, self-efficacy and self-regulatory strategies (action control) for behaviour change. ‘Time’ module encouraged users to reflect on how they spend their time. The ‘Moving More’ module supported users to be more physically active. The ‘Being Social’ module explored the potential benefits of having a meaningful occupation. The ‘Eating Well’ module encouraged the user to consider their current diet and to make changes.
Fillol F, Paris L, Pascal S, Mulliez A, Roques CF, Rousset S, et al. Biomouv SAS Inc, Paris, University of Auvergne, Clermont-Ferrand, 3Biostatistics Unit (Clinical Research and Innovation Direction), University-Hospital Clermont-Ferrand, Human Nutrition Unity, Centre de Recherche en Nutrition Humaine Auvergne, French National Institute for Agriculture, Food and Environment (INRAE), Department of Sport Medicine and Functional Explorations, University-Hospital Clermont-Ferrand, G. Montpied Hospital, Unité fonctionnelle de Recherche Médecine, Clermont University, University of Auvergne, Clermont-Ferrand, Physical and Rehabilitation Medicine, Paul Sabatier University, Toulouse University, Toulouse, France.	September, 2023	Randomized Controlled Trial	*n* = 228 Female = 176 Age > 55y	12-month, prospective, parallel-group, open, multicenter, single-blinded randomized controlled trial (RCT) that enrolled patients attending a 3-week spa therapy treatment. The participants were enrolled in 1 of 8 French spa therapy facilities: Amélie-les-Bains, Bourbon-Lancy, Brides-les-Bains, Le-Boulou, Chaudes-Aigues, Eugénie-les-Bains, Vals-les-Bains, and Vichy.
Quialheiro A, Miranda A, Garcia M Jr, Carvalho AC de, Costa P, Correia-Neves M, et al. Life and Health Sciences Research Institute, School of Medicine, University of Minho; 2ICVS/3B’s, PT Government Associate Laboratory, Braga; 3Department of Physiotherapy, North Polytechnic Institute of Health, CESPU, Famalicão, Associação Centro de Medicina P5, School of Medicine, University of Minho, Braga, Portugal; Office of Infrastructure and Operations in Information Technology, University of Southern of Santa Catarina, Tubarão, Brazil; Regenerative Medicine Center Utrecht, University Medical Centre Utrecht, Utrecht, Netherlands.	February, 2023	Quasi-experimental	*n* = 81 Female = 54 Age > 55y	A design with a nonrandomized allocation of participants in each set of 8 workshops. Sociodemographic, health status, and mobile use information were collected at baseline. Digital and health literacy were measured via the Mobile Device Proficiency Questionnaire and the Health Literacy Scale questionnaires, respectively, at baseline (T1), program completion (T2), and a 1-month follow-up (T3).
Quinn K. Department of Communication, University of Illinois, Chicago, Illinois, USA.	June, 2021	Quasi-experimental	*n* = 36 Female = 25 Age > 65y	Sessions were held in a classroom setting. Six workshop groups met once per week for four weeks, two hours per session (a total of eight classroom hours). Participants used individual laptop computers and visual support was provided via a screen and projector, which was connected to the instructor’s laptop. Each session was led by the researcher, whose research activity is concentrated in social media and social networks.
Zhu, H. and Li, Z. and Lin, W. School of Public Administration School of Emergency Management, Institute of Common Prosperity and National Governance, Jinan University, Guangzhou, China	March, 2023	Observational	*n* = 8505 Female = 4218 Age ≥ 45y	This study uses, in the CHARLS dataset, the CES-D scale to measure older people’s mental health, which is the most widely used tool for measuring depressive symptoms. It investigated how can Internet use improves older people’s health.
Santini S, Fabbietti P, Galassi F, Merizzi A, Kropf J, Hungerländer N and Stara V. Centre for Socio-Economic Research on Aging, IRCCS INRCA-National Institute of Health and Science on Aging, 60,124; Unit of Geriatric Pharmacoepidemiology and Biostatistics, IRCCS INRCA-National Institute of Health and Science on Aging, 60,124; Model of Care and New Technologies, IRCCS INRCA-National Institute of Health and Science on Aging, 60,124 Ancona, Italy; Salumentis OG, 1130; AIT, 1130 Wien, Austria	February, 2023	Quasi-experimental	*n* = 100 Female = 40 Age ≥ 45y 50 in Italy, and 50 in Netherlands, workers aged 55 and over in the transition from work to retirement.	The study followed a mixed-methods design in which both qualitative and quantitative data were collected at three measurement times during the 10-week field trial. It is part of the AgeWell project aimed at co-designing and developing a digital coaching system (hereafter also referred to as Digital Coach-DC) targeting older adults.

Obs.: Austria (AT), Belgium (BE), Bulgaria (BG), Cyprus (CY), Czech Republic (CZ), Germany (DE), Denmark (DK), Estonia (EE), Greece (EL), Spain (ES), Finland (FI), France (FR), Croatia (HR), Hungary (HU), Ireland (IE), Italy (IT), Lithuania (LT), Luxembourg (LU), Latvia (LV), Malta (MT), Netherlands (NL), Poland (PL), Portugal (PT), Romania (RO), Sweden (SE), Slovenia (SI) e Slovakia (SK), United  States of America (US) ,  United Kingdom (UK)

Several studies examined the use of internet-based technologies and digital applications among older adults, focusing on different forms of exposure and engagement. Some studies evaluated the use of digital devices for specific health-related purposes, such as a remote visual acuity assessment tool, while others analysed the role of social media and fitness platforms—including WeChat, WhatsApp, Google Fit, Facebook, and Twitter/X—in health promotion and well-being. In addition, six studies evaluated digital applications designed for desktop and mobile environments, including Digital Human¹, AGE-ON², GET-ON³, LEAP⁴, the Thermactive PA Program⁵, and AgeWell⁶, representing a range of technological approaches from behavioural support tools to systems incorporating elements of artificial intelligence.

**Fig. 2:**
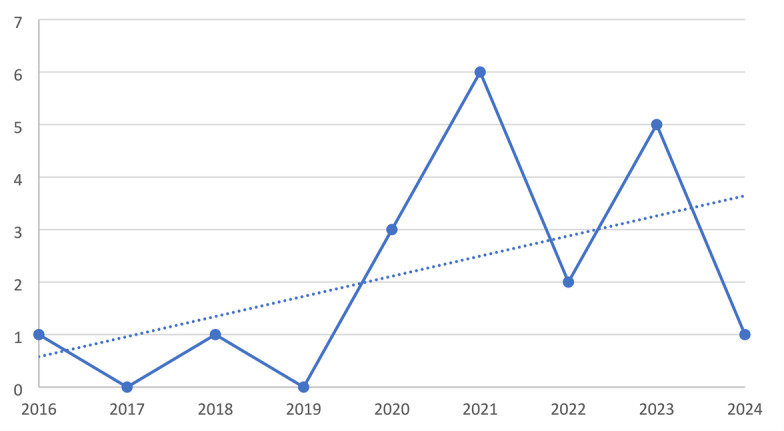
Evolution of the number of publications over the years

No studies directly compared the impact of ICT use before and after the COVID-19 pandemic using an explicit pre- and post-pandemic analytical framework. This absence is consistent with the temporal scope of the review (2016–2024) and with the analytical focus of the included studies, which examined ICT use and related outcomes within specific time windows rather than comparing frequencies or impacts across pre- and post-pandemic periods. Nevertheless, an increase in the number of publications was observed in the years following the onset of the COVID-19 pandemic, reflecting growing research interest in digital health technologies. Figure 2 illustrates the evolution of the number of publications over time.

In response to the research question—*How are digital health applications being used within healthcare services for adults aged 55 years and older?*—and guided by the Population–Concept–Context (PCC) framework, the analysis of the included studies identified three interrelated thematic areas. These concerned (i) the impact of the use of ICTs within healthcare services, (ii) the impact of the use of ICTs on health professionals’ practices, and (iii) the impact of the use of ICTs on the health status and well-being of older adults.

Regarding the impact of the use of ICTs within healthcare services, the majority of the included studies that examined service-level outcomes reported favourable effects associated with the use of digital applications. Five studies indicated that digital tools contributed to relieving pressure on primary healthcare services, for example by supporting self-management or reducing service demand (Braun et al., 2021; Byrne et al., 2021; Jin et al., 2019; Lu et al., 2022; Neil-Sztramko et al., 2020). Among these, three studies also highlighted improvements in the organisation and coordination of social support resources, mental health services, and institutional services, including pharmacies and hospitals.

Additional service-level impacts were identified across several studies. One study reported the use of digital applications to support treatment delivery in nursing home settings (Loveys et al., 2021), while another described their role in promoting community engagement through volunteering (Liu et al., 2020). Other studies indicated that digital applications supported the implementation of health plans and the promotion of healthy retirement trajectories (Lara et al., 2016), encouraged healthy lifestyle behaviours (Fillol et al., 2022; Neil-Sztramko et al., 2020), facilitated links between services and users—particularly in addressing loneliness and supporting treatment engagement (Kamin & Lang, 2020)—and contributed to ongoing health monitoring (Kaminsky et al., 2023) and reductions in missed appointments (Jin et al., 2019). One study also underscored the relevance of digital applications in supporting occupational health services, particularly in the context of health surveillance for older workers (Santini et al., 2023).

By contrast, four of the included studies (10.5%) did not explicitly address the impact of digitalisation on healthcare service delivery, as their primary analytical focus lay elsewhere (Hu & Qian, 2021; Nguyen-Thi et al., 2024; Quialheiro et al., 2023; Quinn, 2021).

In line with the second analytical dimension — the impact of the use of ICTs on health professionals’ practices — the included studies most frequently discussed the use of digital technologies in relation to professional activities and work processes. Ten studies reported positive effects associated with the use of digital applications and internet-based tools, particularly in supporting document management (Kaminsky et al., 2023), training for self-care and professional development (Du et al., 2023; Zhu et al., 2023), medication prescription processes (Jin et al., 2019), and the stimulation of social networking and communication with service users (Quinn, 2021).

Two studies further emphasized that, although digital technologies represent useful and effective tools for healthcare delivery, their successful integration into professional practice depends on continuous training and capacity-building among health professionals (Braun et al., 2021; Fillol et al., 2022). By contrast, one study highlighted potential challenges associated with the introduction of digital tools, noting the risk of increased workload and work-related strain for health professionals if such technologies are implemented without adequate organizational support (Loveys et al., 2021).

Finally, eight of the included studies (42.1%) did not explicitly address impacts on health professionals’ practices, as their primary analytical focus lay elsewhere.

Evidence related to the impact of ICT use on health outcomes among older adults was the most consistently reported across the reviewed studies. Within this analytical dimension, the findings highlighted predominantly positive associations, with a particular emphasis on mental health (Braun et al., 2021; Byrne et al., 2021; Du et al., 2023; Hu & Qian, 2021; Jin et al., 2019; Kamin & Lang, 2020; Lara et al., 2016; Liu et al., 2020; Loveys et al., 2021; Ma et al., 2021; Neil-Sztramko et al., 2020; Quialheiro et al., 2023; Zhu et al., 2023). Reported outcomes included improvements in psychological well-being and resilience, reductions in loneliness and perceived functional dependence, lower risk of depressive symptoms, and enhancements in cognitive and other functional domains.

Beyond mental health outcomes, several studies also reported positive effects related to the biophysiological dimension (Fillol et al., 2022; Jin et al., 2019; Kaminsky et al., 2023; Lara et al., 2016; Lu et al., 2022; Quialheiro et al., 2023; Santini et al., 2023; Zhu et al., 2023). These effects were associated with the use of the internet and digital applications as tools to support self-care practices and the continuity of prescribed therapeutic measures. Examples included digital coaching applications designed to encourage physical activity, such as programmes aimed at promoting exercise for weight management and overall physical health.

**Fig. 3:**
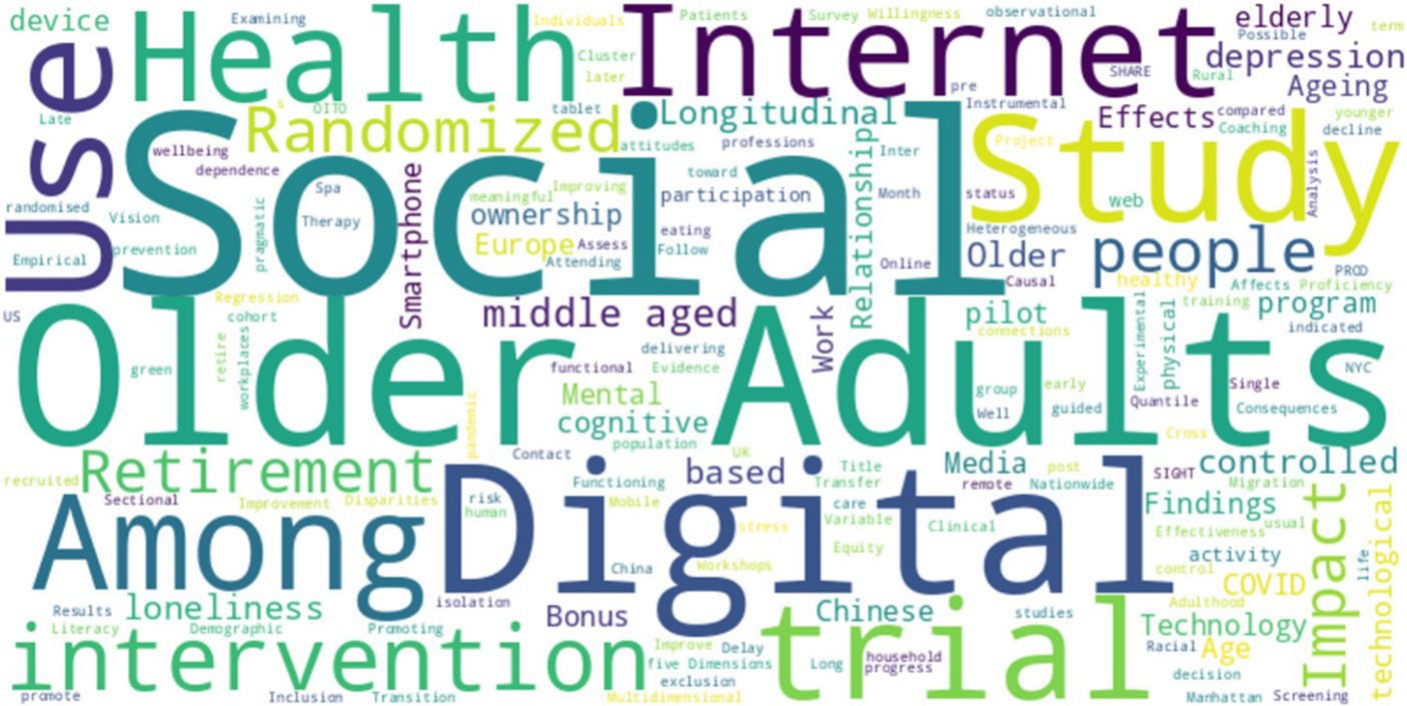
Tagcloud created by ChatGPT 4º based on wordcount of the extraction tool

Additional positive effects were identified within the social dimension (Du et al., 2023; Fillol et al., 2022; Lara et al., 2016; Ma et al., 2021; Quialheiro et al., 2023). These were reflected in a reduced risk of social isolation, sustained engagement in active social life, and strengthened connections with the extended family network. Figure 3 provides an exploratory and illustrative visualization of the distribution of themes related to the social dimension in the reviewed studies. It is a word cloud generated from the repeated terms found across the titles and abstracts of the retrieved literature. The most prominent words represent those that appeared most frequently in the studies. Beyond confirming the relevance of the literature retrieval—visible in the largest terms—the medium‑sized words in the cloud also indicate the geographical origins of the studies, such as China and Europe. Overall, the word cloud highlights that social interaction plays a central role in promoting the health of this population, as reflected in the prominence and clustering of related terms.

In addition to health-related and social outcomes, one study also highlighted financial-related effects associated with job digitalisation (Nguyen-Thi et al., 2024). In this context, the use of digital technologies was linked to reduced physical workload among older workers, contributing to a greater willingness to remain economically active and to sustain financial independence.

Taken together, the reviewed evidence indicates that the use of ICTs may be associated with simultaneous positive effects across multiple life domains. One study reported concurrent benefits spanning health, social, and functional dimensions, illustrating the interconnected nature of these outcomes in the context of digital technology use among older adults (Jin et al., 2019).

## Discussion

The findings of this rapid review provide an overview of how information and communication technologies (ICTs) are being used within healthcare services for adults (aged 55 years and older), highlighting predominantly positive associations across service delivery, professional practice, and individual health outcomes. Rather than demonstrating effectiveness, the reviewed studies collectively suggest ways in which digital health applications are being integrated into healthcare contexts and the types of impacts most frequently reported in the literature.

At the level of healthcare services, the most consistently identified contribution of ICT use relates to the relief of pressure on primary healthcare systems. The reviewed evidence indicates that digital applications may support service delivery by facilitating access to health-related information, enhancing health literacy through reliable digital sources, and enabling closer monitoring and follow-up by health professionals. Together, these mechanisms may contribute to more efficient use of healthcare resources and improved continuity of care, particularly in primary care settings (Andrade & Carvalho, 2023).

The reviewed studies also suggest that the use of ICTs can facilitate coordination and communication across different components of the healthcare system, including hospitals, primary care services, long-term care (such as home care), and other support institutions (e.g. pharmacies and rehabilitation centres). Improved articulation between these services may contribute to more continuous and effective health surveillance for older adults, particularly in contexts where care provision is fragmented. A similar perspective was reported by Castro et al. (2020), who examined the requirements for mobile exercise applications among adults aged 40 to 90 years registered in primary healthcare centres in a low-income and socially vulnerable region of São Paulo State, Brazil. The findings suggest that enhanced digital connectivity between services can reduce pressure on primary care, pharmacies and other support services by improving access to health information, medication management and health resources, while also strengthening quaternary prevention by helping to avoid unnecessary interventions, inappropriate use of resources and practices such as polypharmacy that do not improve health outcomes. (Ferreira et al., 2023).

During the SARS-CoV-2 pandemic, the relevance of ICTs became particularly evident due to the need to maintain access to care for population groups whose increased vulnerability required periods of mandatory isolation. In this context, the implementation of digital technologies was associated with favorable outcomes in terms of health-related support and continuity of care (Clementino et al., 2024). Similarly, Lolich et al. (2022, p. 7), in a study involving home care professionals and stakeholders, highlighted the importance of ICTs in professional practice, identifying their role in “(a) enhancing communication and coordination, (b) supporting both lone workers and isolated clients, and (c)acting as facilitators of social connection.”

The use of ICT also proved to be a positive perspective for health professionals, essentially in the management of care and in the intervention of training for health literacy.

The use of ICTs was also described as a positive development for health professionals, particularly in relation to care management and health literacy training. In the context of care management, however, the literature reveals a certain duality in professionals’ perceptions regarding the use of digital platforms. This is because the computerized recording of care continues to generate ambivalent levels of professional satisfaction. On the one hand, digital applications are regarded as valuable tools for accessing datasets that support diagnostic decision-making, the initiation of therapeutic regimens, and the consultation of large volumes of information (Big Data). On the other hand, some professionals remain reluctant to move away from traditional analogue record systems, due to concerns about potential data breaches, increased workload, and the perceived reduction in time available for direct interaction with users (Rouleau et al., 2017).

The usefulness of ICTs within occupational health services has also been highlighted in the literature, particularly in relation to older workers. Baldwin et al. (2020) emphasize the role of digital technologies in supporting training initiatives aimed at promoting adherence to healthy lifestyles in this population. These findings align with the broader evidence reviewed, which suggest that ICTs may contribute positively to health promotion and preventive strategies across different care settings.

Regarding the use of apps for the training of older users, the data refer to the constitution of a facilitating instrument in the engagement of treatment and its continuity, as well as an important support for caregivers in minimizing their perception of burden (Mikulski et al., 2022). Identical findings were obtained by Brites et al. (2020, p.10) when they found that the use of apps not only facilitated care directed to older users in an outpatient context, but also constituted an important source of support for their caregivers at home to the extent that *“The exchange of messages was highlighted as a way of improving the communication options and social support of the caregiver*.”

Another relevant contribution of apps to the clinical practice of health professionals was related to the intervention in terms of therapeutic prescription, and, according to the results of Wong et al. (2024), the use of these apps will also have exerted a protective effect in mitigating signs and symptoms of loneliness. The results also led to the inference that the use of ICT by health professionals in primary health care has facilitated the intervention to promote healthy lifestyles aimed at older individuals. Other previous studies had already reached the same conclusion, when they found that apps would have been a successful tool in stimulating the practice of physical exercise in older adults (Castro et al., 2020).

Another relevant contribution of digital applications to clinical practice concerns their role in therapeutic management and prescription processes. According to Wong et al. (2024), the use of such applications was associated with a protective effect in mitigating signs and symptoms of loneliness among elderly. In addition, the findings suggest that ICT use by health professionals in primary healthcare settings may facilitate interventions aimed at promoting healthy lifestyles among elderly. These observations are consistent with previous research indicating that digital applications can be effective tools for encouraging physical activity and supporting behaviour change in later life (Castro et al., 2020).

The evidence synthesized in this review also points to positive associations between the use of ICTs and multiple dimensions of health among older adults. Digital applications developed in the field of mental health emerged as the most prevalent ICT-based strategy, underscoring their relevance for health promotion in later life. Beyond their role in cognitive stimulation — which may contribute to delaying cognitive decline — these applications also enable the monitoring of psychoemotional states, potentially supporting the early identification of signs and symptoms associated with dementia (Oliveira et al., 2023). In addition, several studies highlight the contribution of digital tools to dimensions such as self-efficacy and perceived self-esteem, which are particularly relevant in processes of ageing and adaptation to functional change.

Alongside these benefits, the use of digital applications has been associated with reduced perceptions of social isolation and loneliness, as well as lower levels of anxiety and depressive symptoms among older adults (Didoné et al., 2020). Taken together, these findings reinforce the potential of ICT-based interventions to address interconnected mental, emotional, and social aspects of health in later life, while highlighting the need for cautious interpretation given the heterogeneity of study designs and outcomes.

Another area in which digital applications were used for health promotion among older adults concerns the biophysiological dimension. In this context, the selection of app-based strategies appears to be related to their capacity to deliver regular prompts and reminders that support the adoption and maintenance of healthy behaviours, functioning in practice as a form of digital coaching (Abreu & Loureiro, 2023). This approach was illustrated in the study by Evangelista et al. (2024), in which participants were prescribed an application designed to encourage healthy lifestyle practices—such as adherence to a balanced diet, regular physical activity, and weight control—with the aim of reducing cardiocirculatory risk. Additional benefits were reported in interventions led by physiotherapists, where digital applications were used to stimulate functional capacity, contributing to the prevention of falls and potentially delaying the onset of functional dependence among older adults (Moreira et al., 2021).

The association between ICT use and the social dimension of health among older adults was also reflected in predominantly positive terms, particularly with regard to maintaining communication with family members and social networks. In an integrative literature review, Sales et al. (2019) attribute to applications and smartphones an increased perception of accessibility to social benefits, including real-time communication with family and friends and access to social networking platforms that facilitate interaction, information sharing, and knowledge exchange.

At the same time, several challenges and risks have been identified in this context. These include limited access to digital devices, insufficient skills to use them effectively, and broader inequities in digital literacy and access to reliable information sources. Such barriers may contribute to reduced social participation and, in some cases, increase the risk of social distancing and primary social exclusion among older adults (Loureiro et al., 2022).

Finally, research on ICT use in later life has attracted growing interest across multiple disciplines, leading to the development of large-scale, cross-cultural studies focused on older populations (Fan & Yang, 2022; Liu et al., 2020; Santini et al., 2023). While findings from these studies largely emphasize the potential benefits associated with technological advancement, they also underscore the persistence of digital inequalities. Addressing digital illiteracy and unequal access to ICTs remains essential to ensure that the benefits of digital health innovations are equitably distributed. As highlighted by Lee et al. (2024, p.10), “by tackling data absenteeism and technology chauvinism from the outset, it is possible to promote more equitable design and implementation of health applications and wearable technologies for diverse populations”.

However, gaps in the scientific literature — such as the scarcity of topic-specific studies — directly affect the intercultural scope and methodological coherence of the review. Scoping reviews aim to map the breadth and nature of available evidence, identify gaps, and clarify conceptual boundaries across diverse contexts (e.g., populations, settings, cultural frameworks). When the existing literature is limited or highly fragmented, particularly in cultural or socio-geographical domains, the ability of a review to provide a comprehensive intercultural perspective is constrained.

## Conclusion

This rapid review mapped out how information and communication technologies (ICTs) are being used within healthcare services for adults aged 55 years and older, drawing on the Joanna Briggs Institute methodological framework. The synthesis of the reviewed studies identified three interrelated areas of impact: the use of ICTs within healthcare services, their integration into health professionals’ practices, and their association with health and well-being outcomes among older adults.

At the service level, ICTs were consistently described as facilitators of accessibility, continuity of care, and coordination across care settings, particularly in primary healthcare and community-based services. In relation to professional practice, digital technologies were found to support care management, training, and health literacy interventions, while also revealing organisational and workload-related challenges that require careful consideration. At the individual level, the use of ICTs was most frequently associated with outcomes in the mental health domain, alongside benefits related to social participation, functional capacity, and self-care practices.

Taken together, the findings highlight the potential of ICTs to contribute to health promotion and support across multiple dimensions of later life. However, they also underscore the importance of considering sociocultural, environmental, economic, and demographic factors, particularly in urban and socially vulnerable contexts, to avoid reinforcing existing health inequities. Future research should therefore adopt more in-depth and context-sensitive approaches, including longitudinal designs, to better understand the ethical, legal, and equity-related implications of ICT use in care directed at older populations, while respecting cultural diversity and differing levels of digital literacy.

## Electronic Supplementary Material

Below is the link to the electronic supplementary material.

Supplementary Material 1

## Data Availability

The data supporting the findings in this article are available in the Open Science Foundation repository (https://osf.io/). The dataset can be accessed via the following link: https://osf.io/ngdtj/?view_only=e08427ac32024a14be34bcb037da8f85.
